# Antimicrobial Mechanisms and Effectiveness of Graphene and Graphene-Functionalized Biomaterials. A Scope Review

**DOI:** 10.3389/fbioe.2020.00465

**Published:** 2020-05-25

**Authors:** Hiba Mohammed, Ajay Kumar, Elena Bekyarova, Yas Al-Hadeethi, Xixiang Zhang, Mingguang Chen, Mohammad Shahnawaze Ansari, Andrea Cochis, Lia Rimondini

**Affiliations:** ^1^Biomaterials Lab, Department of Health Sciences, Università degli Studi del Piemonte Orientale, Novara, Italy; ^2^Biomaterials Lab, Interdisciplinary Research Center of Autoimmune Diseases, Center for Translational Research on Autoimmune and Allergic Diseases–CAAD, Novara, Italy; ^3^Department of Chemical and Environmental Engineering, University of California, Riverside, Riverside, CA, United States; ^4^Center for Nanoscale Science and Engineering, University of California, Riverside, Riverside, CA, United States; ^5^Department of Physics, King Abdulaziz University, Jeddah, Saudi Arabia; ^6^Advanced Nanofabrication, Imaging and Characterization Core Lab, King Abdullah University of Science and Technology, Thuwal, Saudi Arabia; ^7^Center of Nanotechnology, King Abdulaziz University, Jeddah, Saudi Arabia

**Keywords:** graphene materials, graphene oxide, reduced graphene oxide, nanosheet, antibacterial, biomaterials

## Abstract

Bacterial infections represent nowadays the major reason of biomaterials implant failure, however, most of the available implantable materials do not hold antimicrobial properties, thus requiring antibiotic therapy once the infection occurs. The fast raising of antibiotic-resistant pathogens is making this approach as not more effective, leading to the only solution of device removal and causing devastating consequences for patients. Accordingly, there is a large research about alternative strategies based on the employment of materials holding intrinsic antibacterial properties in order to prevent infections. Between these new strategies, new technologies involving the use of carbon-based materials such as carbon nanotubes, fullerene, graphene and diamond-like carbon shown very promising results. In particular, graphene- and graphene-derived materials (GMs) demonstrated a broad range antibacterial activity toward bacteria, fungi and viruses. These antibacterial activities are attributed mainly to the direct physicochemical interaction between GMs and bacteria that cause a deadly deterioration of cellular components, principally proteins, lipids, and nucleic acids. In fact, GMs hold a high affinity to the membrane proteoglycans where they accumulate leading to membrane damages; similarly, after internalization they can interact with bacteria RNA/DNA hydrogen groups interrupting the replicative stage. Moreover, GMs can indirectly determine bacterial death by activating the inflammatory cascade due to active species generation after entering in the physiological environment. On the opposite, despite these bacteria-targeted activities, GMs have been successfully employed as pro-regenerative materials to favor tissue healing for different tissue engineering purposes. Taken into account these GMs biological properties, this review aims at explaining the antibacterial mechanisms underlying graphene as a promising material applicable in biomedical devices.

## Introduction

Carbon is one of the most important chemical elements that, due to its valency, has atoms that show a considerable capability for binding to other carbon atoms in different manners, exhibiting a variety of allotropes as listed in Table 1. Interestingly, carbon-based materials have emerged as promising principles for a broad range of applications due to their unique mechanical and biological properties. Carbon-based nanostructures (CNSs) such as fullerene, carbon nanotubes (CNTs) and different forms of diamond retain much attention for their wide applications in biological applications such as drug delivery, tissue engineering, imaging diagnosis and cancer therapy (Fisher et al., 2012). These carbon nanostructures have been shown to have potent antibacterial activities toward a broad range of pathogens (Al-Jumaili et al., 2017). Accordingly, their use raised great attention as alternative antibacterial tools. Among the various types of carbon, graphene is considered as a most interesting material due to its unique properties (Ibrahim, 2013; Rojas-Andrade et al., 2017). Over the last few years, research on graphene has significantly increased, due to its physical-chemical properties, which includes strong mechanical strength, large surface area and high resistance to degradation. Graphene active segments and the chemically reactive surface enable graphene tight adhesion to both prokaryotic and eukaryotic cells (Al-Thani et al., 2014) for *in vivo* imaging, diagnosis as well as in the treatment of cancer (Karahan et al., 2018). Moreover, recent literature studies have shown that GMs such as Graphene oxide (GO) and its derivates hold a broad-spectrum antiviral activity toward Virus-like pseudorabies viruses (PRV) and an RNA virus porcine epidemic diarrhea virus (PEDV) (Du et al., 2019).

**TABLE 1:** Chemical bond and dimensionality of carbon allotropes.

Carbon allotrope	Definition	Carbon atom bond	Dimensionality	References
Diamond	Carbon atoms are bonded together in a tetrahedral lattice arrangement;	sp^3^ hybridization	3D	Öhrström and O’Keeffe, 2013
Graphite	Carbon atoms are bonded together in sheets of the hexagonal lattice.	sp^2^ hybridization	3D	Öhrström and O’Keeffe, 2013
Graphene	Consists of a single sheet of graphite.	sp^2^ hybridization	2D	Gurunathan and Kim, 2016
Nano carbons	Carbon atoms are bonded together in hexagonal and pentagonal rings as the basis of an icosahedral symmetry closed-cage structure with different dimensionality: • Fullerenes; referred to as buckyballs or buckminsterfullerenes • carbon nanotubes; single-wall, double-wall and multi-wall	sp^2^ hybridization	0D 1D	Bühl and Hirsch, 2001; Yadav and Ritesh, 2008
Graphenylene (biphenylene carbon)	Carbon atoms are bonded together in a hexagonal lattice based on biphenylene-like subunits.	sp^2^ hybridization	2D	Lüder et al., 2016

Graphene is precisely defined as single carbon layer of the graphite structure (Fitzer et al., 1995; Geim and Novoselov, 2007). This monolayer is composed of sp^2^-hybridized carbon atoms bonded to each other with a 0.142 nm length bond (Dasari et al., 2017) and tightly packed into a honeycomb lattice, forming a two-dimensional crystal (Novoselov, 2004). The interest in this material escalated in 2004 when Nobel laureates Andre Geim and Konstantin Novoselov published the deposition and characterization of graphene on solid supports (Novoselov, 2004). Since the inception of these experiments, researchers around the world have continuously explored the excellent physical and mechanical properties of this “miracle or wonder material” (Novoselov et al., 2012; Edwards and Coleman, 2013; Eigler and Hirsch, 2014). Graphene exhibits remarkably high mechanical stiffness (Berger et al., 2006; Novoselov et al., 2012; Edwards and Coleman, 2013; Suk et al., 2013), exceptional electronic transport properties (Zhang et al., 2005; Berger et al., 2006; Miao et al., 2007; Bunch et al., 2008; Nair et al., 2008; Loh et al., 2010; Balandin, 2011), good thermal conductivity (Balandin, 2011; Suk et al., 2013), high surface area (Nair et al., 2008), desirable elastic properties (Zhang et al., 2005) and gas impermeability (Bunch et al., 2008). The chemical inertness and the presence of free π electrons make graphene as a promising carrier for controlled drug delivery (Loh et al., 2010). In general, all these properties enable the application of graphene in the fields of energy storage devices (Xu et al., 2013), sensors (Wu et al., 2013), fuel cells (Chen Y. et al., 2013), solar cells, electronics (Hu et al., 2010), and high-strength materials (Lee et al., 2013).

In the biomedical field, graphene demonstrates impressive properties in diagnosis (Castillo et al., 2013; Chen J.Y. et al., 2013; Hu et al., 2013; Kwon et al., 2013; Wang et al., 2013b) and has therapeutic potential as a nanocarrier and drug delivery vehicle (Gonçalves et al., 2013; Wang Y. et al., 2014; Vinothini and Rajan, 2017). Graphene and its derivatives also demonstrate a valuable impact in tissue engineering and exhibit strict antimicrobial activities; these capabilities render them suitable candidates for fabricating nanohybrid structures applicable in various biomedical fields such as tissue differentiation, regeneration and infection control (Shang et al., 2019). Graphene nanohybrids have been fabricated as potentially effective dressing scaffolds aimed at wound healing. The idea of such nanohybrid scaffolds relied on the synergistic effects of graphene for infection control as well as its regenerative capacity (Shang et al., 2019). The high antibacterial capacity is mainly associated with the physical damages occurred upon direct contact to bacterial membranes by the sharp edges of graphene sheets, while the regenerative impact is based on the scaffold potential to promote the adhesion and proliferation of mesenchymal stem cells (MSCs) (Vinothini and Rajan, 2017). Many research works have revealed the efficacy of graphene against both gram-positive and gram-negative bacteria depending on a variety of mechanisms and factors related to both the bacterial components and the nanoparticles themselves (Al-Thani et al., 2014; Karahan et al., 2018). This Review article offers a detailed discussion of the antibacterial activities of graphene, graphene derivatives, and graphene nanocomposites.

### Graphene Derivatives

Despite graphene’s sophisticated properties, it exhibits some limitations in certain applications that require definite characteristics (Dreyer et al., 2010). One of the main limitations is graphene’s tendency to agglomerate due to its very low water dispersibility as a hydrophobic material in addition to its large surface area and high surface energy (Dreyer et al., 2010; Yang et al., 2014). To improve graphene’s properties, chemical modifications were considered valuable approaches resulting in graphene derivatives which are applicable in different fields (Romero et al., 2017). Some examples are reported below.

#### Graphene Oxide

Graphene oxide (GO) is the oxidized derivative of a graphene molecule, obtained by acid oxidation of graphite (Dreyer et al., 2010), i.e., it contains oxygen functional groups (hydroxyl, carboxyl, carbonyl, and epoxy). Thus, GO is an extremely hydrophilic molecule which is significantly beneficial in power-harvesting and electronic applications (Romero et al., 2017). The extreme hydrophilic properties of GO render these molecules insoluble in organic solvents such as alcohol, toluene, etc. (Romero et al., 2017). Moreover, GO is an amorphous molecule with many defects that weaken its mechanical characteristics, rendering them much weaker than those of pristine graphene or reduced graphene oxide (Dreyer et al., 2010). Thus, GO is chemically or thermally reduced to partially restore the properties and structure of graphene; the generated material is referred as reduced graphene oxide, rGO (Dreyer et al., 2010; Pei and Cheng, 2012).

#### Reduced Graphene Oxide (rGO)

Reduced graphene oxide (rGO) is a graphene derivative obtained through chemical or thermal reduction of GO, i.e., the reduction of the oxygen functional groups in GO. rGO is characterized by its moderately reduced number of functional groups, a high number of electroactive sites, and its structural similarity to graphene (Báez et al., 2017). Moreover, it exhibits a large surface area, high reactivity and biocompatibility (Wang and Shi, 2015; Báez et al., 2017). rGO demonstrates properties of both pristine graphene (high surface area and strength) and GO (moderate dispersibility in water) (Singh et al., 2011; Lv et al., 2013; Babak et al., 2014; Pan et al., 2015; Sharma and Kothiyal, 2015; Lu et al., 2016; Zheng et al., 2017). Therefore, graphene characteristics are partially restored through rGO generations (Dreyer et al., 2010).

### Antimicrobial Activity

Progressive advances in the nanoscience has opened the door to a considerable number of nanomaterial applications in a variety of fields. Interestingly, graphene material (GM) and its derivatives occupy a significant area of the contemporary applications with a significant role in the biological and medical domains, especially due to their nanomaterial-triggered biosensing action and direct interaction with various cell types and statuses such as bacteria, fungi, and tumoral/normal mammalian cells (Liu S. et al., 2011; Akhavan et al., 2012; Al-Thani et al., 2014). Inspired by GMs’ properties in thermotics, electronics, optics, and mechanics, in addition to their unique structure, much effort has been made to uncover their broader therapeutic roles (Hu et al., 2010; Krishnamoorthy et al., 2012) not only in diagnosis and treatment but also in microbial infections (Zou et al., 2016).

Despite the progress made in investigating GMs’ antimicrobial activity, the underlying antimicrobial mechanisms remain controversial (Zou et al., 2016). However, several recent experimental outcomes have suggested that the physicochemical characteristics of GMs, such as particle size, morphology, and surface functionality (Dallavalle et al., 2015; Mangadlao et al., 2015; Perreault et al., 2015a) play a pivotal role in the predominant mechanisms including oxidative stress, nanoknives, and wrapping/trapping (Akhavan et al., 2011; Li et al., 2015; Pham et al., 2015).

The research findings of this review which involve the antibacterial activity of graphene materials were obtained through MEDLINE and Scopus databases using the following keywords: graphene, reduced graphene oxide, graphene nanohybrids AND antibacterial activity. Based on our research findings, this review aims to illustrate the possible mechanisms that might influence the GMs’ antibacterial activities.

### Carbon-Based Materials

Carbon nanostructures (CNSs) such as fullerene and carbon nanotubes (CNTs) have been shown to hold strong antibacterial activities toward a broad-rang of pathogens (Al-Jumaili et al., 2017).

The mechanism by which CNSs kills bacteria is complex and depends on different properties such as composition and surface physical-chemical modifications. Moreover, the properties of the target microorganisms and the characteristics of the environment in which cell CNS interactions take place can also represent an important factor.

Fullerenes antibacterial activity takes place by the physical interaction between fullerenes and the outer cell membrane (Lyon et al., 2008). Where fullerene tightly binds the lipidic portion of the cell membrane, it determines wall disruption and DNA cleavage after internalization due to its high surface hydrophobicity that strongly interact with membrane lipids. Due to the different percentage of membrane lipids between Gram-positive and Gram-negative bacteria, most of the studies demonstrated that fullerene was more active toward Gram-positive species. Moreover, it seems to be light-sensitive because of a powerful antibacterial activity toward many bacterial species when exposed to light (Brunet et al., 2009). This can be due to the unique fullerene shape: in fact, it looks like a cage that is able to absorb light through π electrons causing reactive oxygen species release (Maas, 2016).

Carbon nanotubes (CNTs) are described as a hollow structure formed by rolled graphene sheets. Moreover, CNTs can be categorized as single-walled nanotubes (SWNTs) and multi-walled nanotubes (MWNTs) (Kang et al., 2008).

SWNTs have a higher antibacterial effect than most of carbon nanomaterials. This can be due to their small size that enables membrane perturbation. The mechanism of CNTs is particularly affected by several factors like surface functional group, surface chemistry, diameter, length and electronic structure. In particular, nanotubes length seems to play an important role during the interaction of the cell membrane even if the exact mechanism by which CNTs interact with cell wall is still unclear (Kang et al., 2007).

The two-dimensional graphene exhibits distinct physicochemical characteristics which depend on the synthetic method (Liu et al., 2012; Song et al., 2015; Zou et al., 2016). With the application of certain synthetic approaches such as chemical vapour deposition (CVD) and epitaxial growth methods, a flawless crystal graphene structure is obtained while the implementation of other synthetic methods such as chemical exfoliation, mechanical cleavage, and chemical synthesis generate structurally defective graphene materials which in turn influence their physicochemical properties (Hummers and Offeman, 1958; Novoselov et al., 2005; Hao et al., 2013).

Interestingly, the presence of different defects within graphene – such as basal plane destruction and oxygen-containing groups – creates more active sites for enhanced interaction with various ions, molecules, and materials (Ameen et al., 2013; Huang et al., 2014; Zhang et al., 2015; Zou et al., 2016). Furthermore, these defects modify the intrinsic characteristics of GMs such as morphology, layer number, lateral size, and dispersibility (Zhang et al., 2009, 2014; Wang X. et al., 2011; Liu R. et al., 2011). Based on the continuously increasing experimental and theoretical reports, a close relevance exists between the antibacterial efficacy and the physicochemical and/or structural characteristics of GMs (Hu et al., 2010; Krishnamoorthy et al., 2012; Tu et al., 2013; Hui et al., 2014; Li et al., 2015; Pham et al., 2015).

## Physical-Chemical Properties Influencing Graphene Antibacterial Activity

### Lateral Size

**FIGURE 1:**
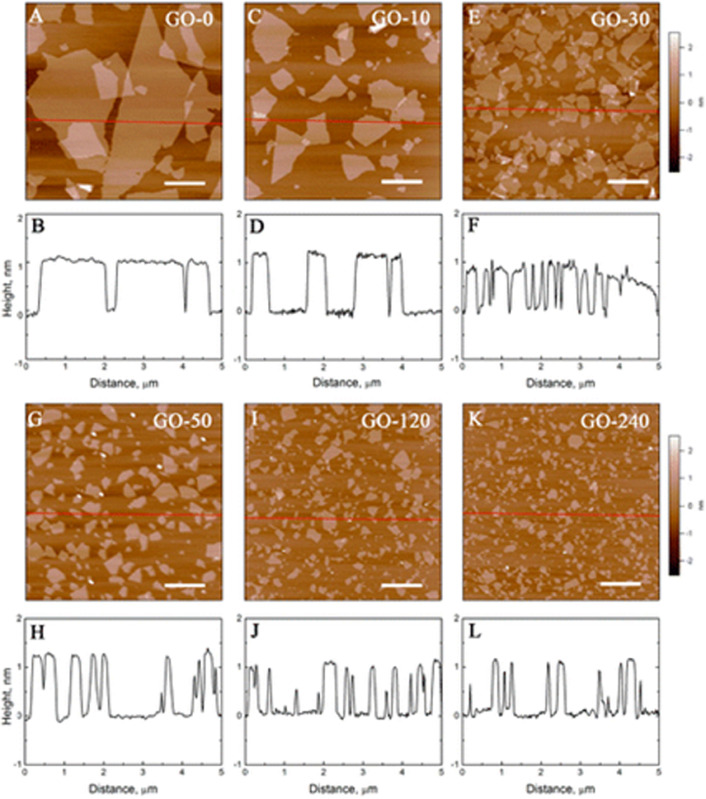
AFM height images of GO sheets dried on mica surface after tip sonication for 0 **(A)**, 10 **(C)**, 30 **(E)**, 50 **(G)**, 120 **(I)**, and 240 min **(K)**. All scale bars are at 1 μm. The corresponding height profiles along red lines in AFM images: 0 **(B)**, 10 **(D)**, 30 **(F)**, 50 **(H)**, 120 **(J)**, and 240 min **(L)**. Reproduced with permission from Perreault et al. (2015a).

Lateral size is a crucial determinant of GMs’ antimicrobial effectiveness and it can be modified based on the synthetic method or post-treatment (Figure 1). The GM’s adsorption, dispersion, and sharp edges are hugely affected by the particle size; these properties are, in turn, pivotal to the GMs’/microorganism’s physicochemical interaction (Cai et al., 2011). The larger the lateral size of the GM, the stronger the adsorption ability, which is attributed to the higher surface energies (Zou et al., 2016). An investigative study stated that antimicrobial effects were stronger in association with larger-sized GO sheets than with smaller sheets (Liu et al., 2012). The lateral size is generally affected by the synthesis method and defects are always present in the GMs prepared by the redox method. Meanwhile, more defects are associated with a decreased lateral size of the produced GMs (Zou et al., 2016). To that end, the findings obtained by Perreault et al. (2015a) demonstrate that the greater the defects in smaller GO nanosheets, the stronger the antimicrobial activity against *Escherichia coli (E. coli).*

### The Number of Layers

**FIGURE 2:**
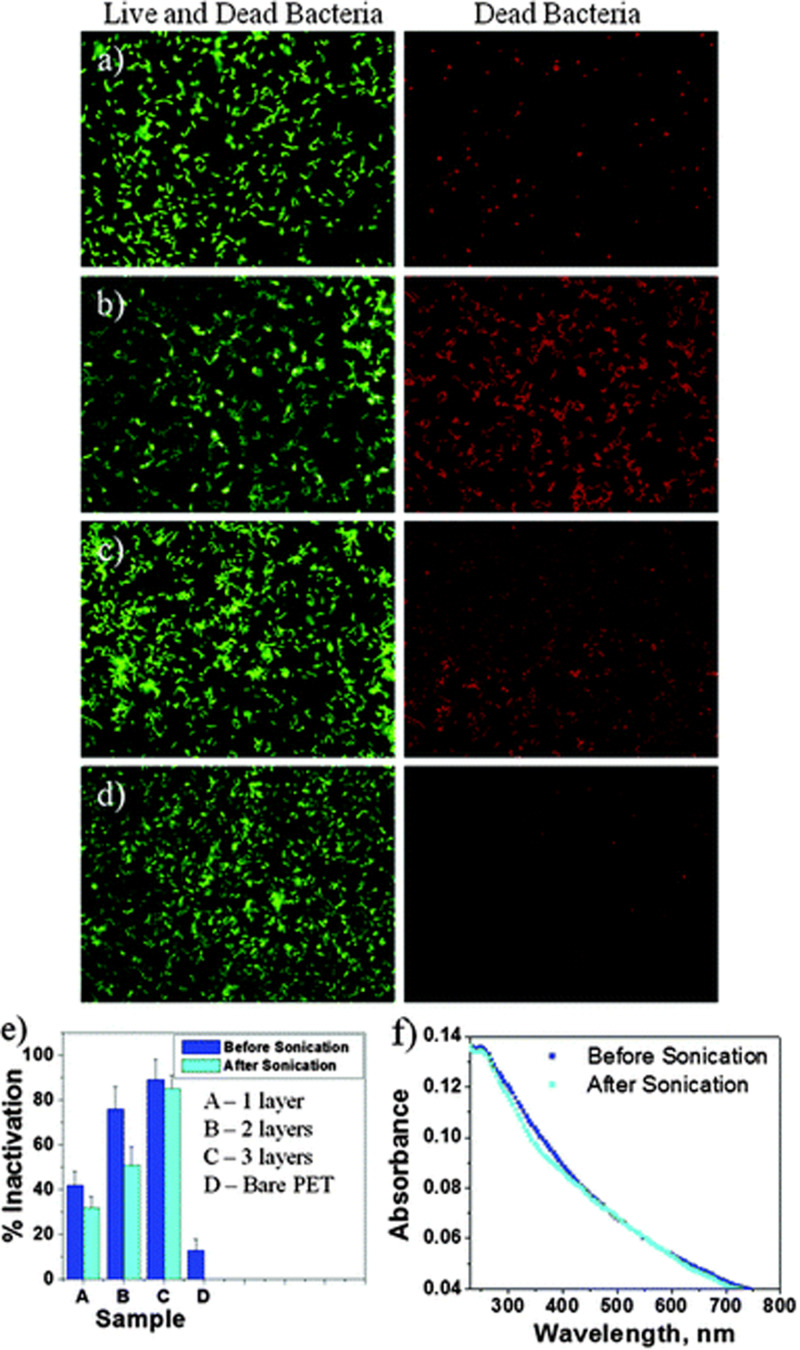
Representative fluorescence images of *E. coli* on a single **(a)**, double **(b)**, and triple **(c)** layers of GO-LB and bare PET **(d)**. **(e)** Comparison of the antibacterial effect before and after ultrasonication. **(f)** UV-Vis absorbance of 3-layer GO-LB film before and after ultrasonication. Reproduced with permission from Akhavan et al. (2012).

The number of graphene layers is an important determinant of its antimicrobial activity; i.e., increased GMs’ layers increase the thickness, causing a weakened “nano knife” effect, decreased dispersibility, and increased aggregation tendency, resulting in reduced contact between GMs and microorganisms (Zou et al., 2016). Wang et al. (2013a) demonstrated, through theoretical results, that the energy barrier for three-layer graphene sheets with nooks to penetrate the lipid bilayer is superior to that for monolayer sheets of the same lateral size. This might indicate that few-layer graphene sheets have an intensive capacity to damage the bacterial cell membrane (Zou et al., 2016). On the other hand, the experimental results obtained by Mangadlao et al. demonstrated that an increased number of GO-sheets resulted in a stronger antimicrobial effect against *E. coli* as illustrated in Figure 2 (Mangadlao et al., 2015). This finding has been interpreted as follows: The number of layers influences the surface properties which induce the basal plane antimicrobial activity, i.e., both the edges and surface of GMs play key roles in antimicrobial activity (Zou et al., 2016).

### Particles Shape

**FIGURE 3:**
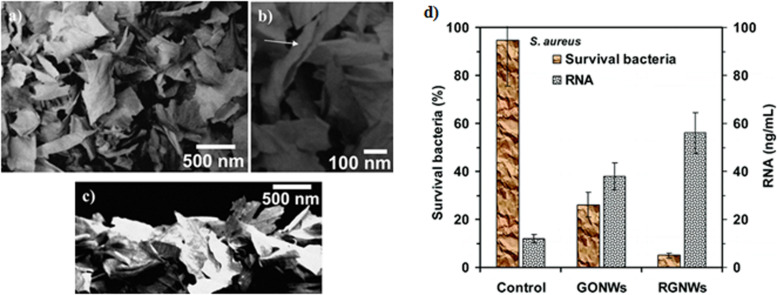
Antimicrobial activity of graphene oxide nanowalls (GONWs) and reduced graphene oxide nanowalls (RGNWs). **(a–c)** SEM images of **(a)** the GONWs deposited on stainless steel substrate by electrophoretic deposition, **(b)** the nanowalls at higher magnification showing those are nearly perpendicular to the substrate, and **(c)** the cross-sectional view of the nanowalls. **(d)** Cytotoxicity of GONWs and RGNWs to *S. aureus*, and concentrations of RNA in the PBS of the *S. aureus* bacteria exposed to the nanowalls. Reproduced with permission from Ameen et al. (2013).

The antimicrobial activity of nanoparticles is considerably influenced by the particle shape. Previous studies have demonstrated the shape-dependent cytotoxicity of both carbon nanotubes (CNTs) and silver nanoparticles (AgNPs) (Poland et al., 2008; Sadeghi et al., 2012). Moreover, theoretical simulation states that nanoparticle shapes are essential for their interaction with the lipid bilayer in a translocation process (Yang and Ma, 2010). An interesting finding demonstrated that the sharp edges of GO nanowalls (GONWs) and rGO nanowalls (RGNWs) significantly decreased the rate of survival of both *Staphylococcus aureus* (*S. aureus*) and *E. coli* (Akhavan and Ghaderi, 2010) as illustrated in Figure 3.

Additionally, experimental verification of particle shape efficacy was carried out through the use of graphene films with different topography on both aspects to observe the effects of *S. aureus* and *Pseudomonas aeruginosa (P. aeruginosa*) (Al-Thani et al., 2014). Interestingly, the smooth-top-side graphene film presented efficient bactericidal activity against both round-shape *S. aureus* and *P. aeruginosa*, while the rough-bottom-side graphene film was effective in deactivating only rod-shaped *P. aeruginosa* (Zou et al., 2016). This antimicrobial activity might be attributed to the easy permeation of graphene nanoparticles into the cell membrane due to the low energy barrier of these sharp-corner protruded particles (Li et al., 2013).

### Surface Modifications

**FIGURE 4:**
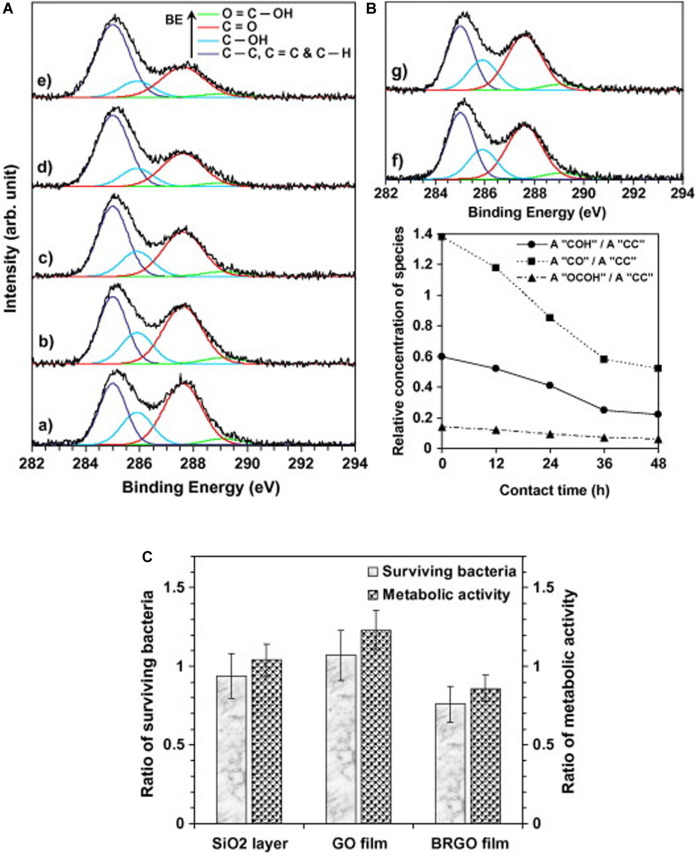
Effective reduction of graphene oxide (GO) with bacteria produces bacteria-reduced GO (BRGO). Peak deconvolution of C(1s) core level of XPS of the graphene (oxide) sheets: (a) before exposure to the bacteria, and after exposure to the bacteria for (b) 12 h, (c) 24 h, (d) 36 h, and (e) 48 h in panel **(A)**, and after exposure to (f) only the culture medium of the bacteria (without the bacteria) and (g) the culture medium containing the bacteria but without any glucose, for 48 h in panel **(B)**. **(C)** Shows the peak area **(A)** ratios of the oxygen-containing bonds to the C-C bonds (obtained by XPS) vs. contact time of the bacteria to the sheets. **(C)** Bioactivity of the *E. coli* bacteria on surfaces of the bare SiO2 substrate, GO and BRGO sheets at room temperature after 2 h. Reproduced with permission from Zhang et al. (2014).

The contact and interaction between GMs and other molecules, such as other materials, proteins, DNA/RNA, lipids, etc., are essential for their antimicrobial activity. The agglomeration tendency of pristine graphene potentially decreases its contact with other particles (Zou et al., 2016). Thus, the modification of graphene’s surface or edge characteristics via covalent and non-covalent modulation has been found to play a key role in preventing particle agglomeration and, consequently, influencing their antimicrobial activities (Li et al., 2008; Park et al., 2010). An interesting study demonstrated that rGO exhibited stronger antimicrobial activity against *S. aureus* and *E. coli* than GO did (Ameen et al., 2013; Figure 3). Another study demonstrated that *E. coli* proliferation was inhibited by rGO, whereas no cytotoxicity was observed in association with GO (Akhavan and Ghaderi, 2012), as shown in Figure 4.

This variation between rGO and GO is related mainly to the surface modulation and surface properties. In this respect, studies have presented that GMs’ antimicrobial efficacy might be enhanced by the impact of covalent modulation with oxygen-containing groups. The oxygen groups can influence GMs’ amphipathic and blade effect, which subsequently modifies their antimicrobial activities (Balandin et al., 2008; Yadav et al., 2013; Fan et al., 2014; Hui et al., 2014; Musico et al., 2014; Tian et al., 2014). In conclusion, GMs can influence microorganism survival through the adsorption interaction between GMs and other materials, ions, and molecules. This interaction modifies the microenvironment of bacteria or other microorganisms, thereby inhibiting their proliferation (Zou et al., 2016).

### Agglomeration and Dispersion

Due to the high surface energies of GMs (including CNTs, graphene and rGO), these particles are predisposed to agglomeration. This property modulates the edge and surface characteristics of the nanoparticles, which in turn alters their antimicrobial activities (Wick et al., 2007). Regarding CNTs, one of the primary factors directing their antimicrobial activities is the agglomeration tendency, which causes a reduced surface area and shapes alteration of the nanomaterials (Wick et al., 2007). GMs’ agglomeration weakens their dispersibility and adsorption capacity, which alters blade efficacy and consequently reduces their interaction with the microorganisms (Zou et al., 2016). However, these properties differ among the different forms of graphene, with GO dispersion exhibiting the most potent antimicrobial activity against *E. coli*, followed by rGO, graphite (Gt), and graphite oxide, successively (Liu S. et al., 2011). These findings were interpreted as different dispersion conjunctures of the mentioned nanomaterial, i.e., the proper dispersion of GO results in thin sheets of this nanomaterial which is capable of easily wrapping bacteria, whereas rGO exhibits aggregate formation and reduced antimicrobial impact when it is not fully exfoliated (Zou et al., 2016). On the other hand, the results obtained by Akhavan et al. show that rGO is more potent than GO in bacterial inactivation; this is attributed to *E. coli* trapping and its ability to gradually wrap bacteria during the formation of rGO aggregates in the suspension (Akhavan et al., 2011).

## GMs Antimicrobial Activities

A variety of experimental circumstances should be considered when assessing the antimicrobial activities of GMs. These include the state of the applied material, the bacterial type (aerobic/anaerobic), the implemented ambiance (*in vitro*/*in vivo*), and the microorganism genera such as the shape (rod/round) and category (Gram-positive/Gram-negative). Each microorganism has its capacity for growth under certain physicochemical circumstances; thus, it is important to realize and grasp these conditions to control microorganism growth in a definite and clear manner (Zou et al., 2016). For example, an experimental study carried out by Pham et al. (2015) found that the antimicrobial activity of the rough surface of graphene films was stronger against *P. aeruginosa* than against *S. aureus*, with 87.6 and 43.1% of these bacteria killed, respectively. These results were interpreted as indicating the extent to which the antimicrobial impact is extremely dependent on the selected bacterial species; some examples are given in Table 2.

**TABLE 2:** Antibacterial activity of GMs concerning experimental surroundings.

GMs	Experimental surrounding	Type of experimental surrounding	Impact	References
GO	Luria-Bertani	Nutrient broth	*E. coli* growth enhancement	Zhou, 2004
GO, rGO	Papers and suspension	Material state	*E. coli* inactivation	Akhavan et al., 2011
GO	Luria-Bertani, bovine serum albumin (BSA), L-tryptophan (TrP) added to the saline	Nutrient broth	Inhibition of the antimicrobial activity	Fan et al., 2014
GO	Impurities such as manganese and sulfur	Hummer’s synthesis method	Carboxyl and hydroxyl groups of GO detach, causing low pH, which disturbs the microorganism microenvironments and influences the dispersibility of GMs	Liu et al., 2012; Dallavalle et al., 2015

### Direct Contact Mechanisms

During the primary bacterial-antibacterial agent interaction, bacterial cell death is induced via the inhibition of essential bacterial cell functions (Zou et al., 2016). These antimicrobials involve mainly the bacterial components or systems, inhibiting cellular growth (bacteriostatic) or inducing cell death (bactericidal). These antibacterial activities are attributed mainly to the physicochemical interaction caused by deteriorated cellular components, principally proteins, lipids, and nucleic acids (DNA/RNA) (Zou et al., 2016). It is well known that bacterial cell form and rigidity are tightly linked to the peptidoglycan proteins and the lipid bilayer that constitute the principal cell wall/membrane components. Thus, bacterial cell death can be induced by any interference with peptidoglycan precursor assembly resulting in a weakened and, consequently, collapsed cell wall. Moreover, nucleic acid damage results in the inhibition of microorganism duplication. Interestingly, GMs, as capable antimicrobials, can exhibit both bacteriostatic and bactericidal activities by interfering with bacterial lipids, proteins, and nucleic acids through electrostatic adsorption, hydrogen bonding, and π-π stacking (Akhavan and Ghaderi, 2010; Li et al., 2013, 2015; Tu et al., 2013). This interaction might induce lipid extraction, protein disruption, nanoknives, or reactive oxygen species (ROS) mechanisms, inhibiting or killing the cell (Zou et al., 2016).

### Interaction With Bacterial DNA/RNA

**FIGURE 5:**
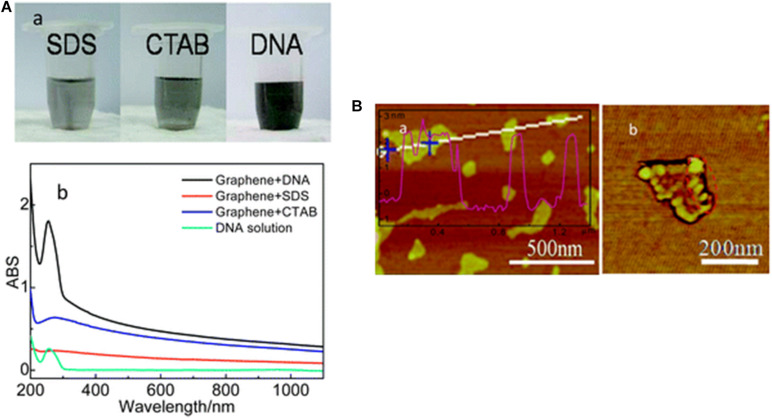
Adsorption of DNA on graphene. **(A)** Hybridization of graphene with ss-DNA strands. (a) digital photos of graphene suspensions with different dispersants (SDS, CTAB and DNA); (b) UV-vis spectra of DNA aqueous solution and graphene suspensions. **(B)** AFM images of graphene-DNA GN/DNA hybrids. (a) AFM image with a cross-section contour and (b) a phase image of a single GN/DNA sheet. Reproduced with permission from Li et al. (2013).

Bacterial genomes consist of a single circular molecule called bacterial chromosome (double-stranded DNA). Besides, bacteria often contain small extrachromosomal circular DNA molecules named plasmids. The antibiotic-resistant genes or virulence factors are contained within the genome. Thus, when the bacterial DNA is exposed to corruption or malfunction during its replication, the bacterial cell undergoes mutation or dies. Once DNA/RNA is exposed to GMs, interactions with these nanoparticles may occur through hydrogen bonding, π-π stacking, and electrostatic adsorption due to the presence of oxygen- and nitrogen-containing groups in addition to the π-conjugated structure (He et al., 2010; Lv et al., 2010). The GMs’ permeation into the microorganism alters the DNA/RNA structures and properties (He et al., 2010; Lv et al., 2010), leading to inactivation or death of the microorganism (He et al., 2010; Gurunathan et al., 2012). Several experimental findings have proven the strong bacterial DNA-GMs’ interactions through the application of DNA/RNA-coated GMs in molecule recognition (He et al., 2010; Lv et al., 2010), DNA translocation and sequencing (He et al., 2011; Banerjee et al., 2015), and anticancer drug delivery (Kim et al., 2015; Mo et al., 2015). A graphical example of graphene interaction with DNA is given in Figure 5.

### GMs’ Interaction With Bacterial Proteins

**FIGURE 6:**
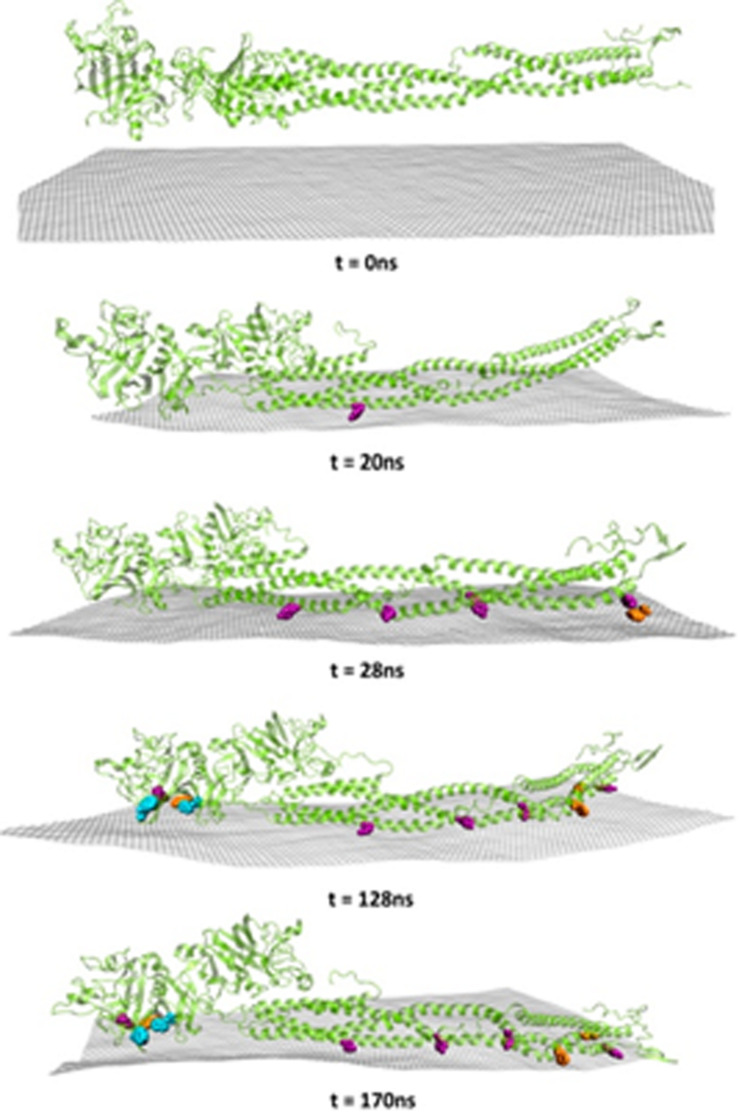
Molecular dynamics snapshots of the absorption of bovine fibrinogen onto graphene. Cartoon representations of the full protein are depicted in yellow, and hydrophobic Tyr (purple), Phe (orange), and Trp (blue) within 0.5 nm distance of the graphene surface are represented as van der Waals spheres. Atoms corresponding to the graphene sheet are colored in gray. Reproduced with permission from Yadav et al. (2013).

Cellular proteins are generally divided into functional and structural proteins. Functional proteins are responsible for metabolism regulation, while structural proteins act as building units for different cellular components. Proteins are found in the cell wall, cell membrane, and cytoplasm and are composed of amino acids (nitrogen-containing groups) that can drive the hydrogen- bonding interactions with a variety of substances. Besides, the π-conjugated structures that exist in some amino acids can induce interaction with other substances that contain π-conjugated structures via π-π stacking. Moreover, under certain conditions, amino acids demonstrate various electronegativities. GMs are characterized by large π-conjugated structures and an abundance of oxygen-containing groups; thus, strong interactions of GMs with cellular proteins have frequently been reported (Zou et al., 2016). To investigate graphene-protein interaction, a graphene-modified Si/SiO_2_ device was designed by Alva and coworkers, who found that the direct adsorption of protein on graphene leads to immediate protein denaturation (Alava et al., 2013). On the other hand, the protein might also influence the properties and antimicrobial action of GMs. Accordingly, Chong et al. (2015) investigated the interactions between different GMs and four proteins and concluded that both GO and rGO were not cytotoxic when coated with proteins via hydrophobic or π-π stacking interactions. The π-π stacking interactions occur through the aromatic rings corresponding to Trp, Tyr, and Phe which align with the graphene surface (Chong et al., 2015; Figure 6).

### GMs’ Interaction With Phospholipids

The phospholipid bilayer consists of the basal plane of the cell membrane and acts as a barrier, keeping proteins, ions, and different molecules in place as well as protecting the cell from damage. Destruction of the phospholipid bilayer in microbial cells was found to be a key factor in the microorganism’s death (Li et al., 2013). As mentioned previously, GMs comprise large π-conjugated structures and exhibit extreme hydrophobic properties. Once GMs contact the microorganism, a hydrophobic interaction called “nanoscale dewetting” takes place between the GMs and phospholipid molecules at the cell membrane to forcibly extract the lipid bilayers onto the GMs’ surfaces and drive the collapse of the cell membrane (Zhou, 2004; Liu et al., 2005; Berne et al., 2009). As a proof, Tu et al. (2013) proved, by theoretical and experimental results, that GO exerts a destructive extraction of phospholipids from *E. coli* and reduces its viability via potent dispersion interaction between GO and the cell membrane of *E. coli.*

**FIGURE 7:**
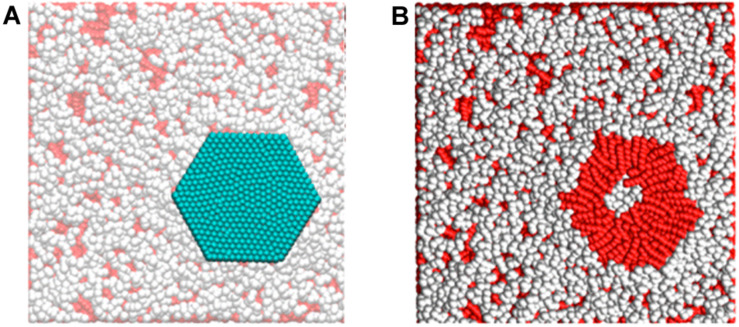
**(A)** Sheet adhering to the phospholipid membrane. **(B)** Peeling off the sheet shows that the hydrophobic tails directly interact with hydrophobic graphene. Reproduced with permission from Liu S. et al. (2011).

Moreover, Dallavalle et al. (2015) used theoretical modeling to shed light into the interactions between graphene and phospholipids. Figure 7 shows that the phospholipids directly under the graphene interacted with the sheet with the hydrophobic tail and these hydrophobic–hydrophobic interactions allowed the graphene sheet to adhere to the membrane.

Furthermore, this study revealed that the hydrophobic interaction enables the small graphene sheets to penetrate the phospholipid bilayer while forcing the larger nanosheets to lie on the cell membrane surface, interrupting the phospholipid molecules’ interactions. Another important finding demonstrated by Li et al. (2013) revealed that micrometer-scale graphene sheets with sharp edges and protruding corners are capable of easily permeating the cell membrane via coping the decreased energy barrier that the powerful hydrophobic interactions generated.

## Mechanisms of Graphene-Mediated Antimicrobial Activities

Despite the substantial effort devoted to uncovering the exact mechanism of graphene antimicrobial activity, the experimental findings are still controversial, and a universal mechanism remains to be established. As mentioned previously, the physicochemical interaction between GMs and microorganisms can drive their antimicrobial activity. Accordingly, three principal mechanisms have been proposed; (i) nanoknives through the sharp GM edges (cell membrane stress) (Akhavan and Ghaderi, 2010; Liu S. et al., 2011), (ii) oxidative stress with/without ROS production (West and Marnett, 2006; Li et al., 2015), or (iii) wrapping or trapping the bacterial cell membrane by the flexible thin-film structure of GMs (wrapping mediated blockage of membrane transport) (Mejías Carpio et al., 2012; Liu et al., 2012).

### Nanoknives’ Action of Sharp-Edged GMs by Cell Membrane Stress

As many cumulative studies have documented, the antimicrobial impact of GMs is critically influenced by the nanoknives’ action, which is related to the GMs’ sharp edges, which resemble blades or cutters. The nanoknives’ mechanism of action is referred to as “penetration mode” or “insertion mode” and has been interpreted according to theoretical simulations and experimental results as “the intrusion of the blade-like GMs into the microbial cell membrane with the consequent leakage of the cytoplasmic content and cell death (Zou et al., 2016). In this context, Akhavan and Ghaderi (2010) discovered that direct contact between the extremely sharp edges of both GONWs and rGONWs with the bacteria *S. aureus* and *E. coli* destroys the bacterial cell membrane as well as RNA leakage and, subsequently, bacterial death. Supporting this, many other research findings indicate the leakage of intracellular content, including DNA/RNA, as a result of the mechanical disruption of the cell membrane-derived by sharp-edge GM contact (Chen J. et al., 2013, 2014; Wang et al., 2013c; Musico et al., 2014; He et al., 2015). Moreover, Liu S. et al. (2011) investigated the impact of GMs on *E. coli* and concluded that sharp edges act as cutters that induce cell membrane stress, resembling the cytotoxic effect caused by single-walled CNTs (Kang et al., 2007). Another significant parameter influencing the antimicrobial activity of GMs could be edge density; this suggestion was recently introduced by Pham et al. (2015), who designed graphene nanosheets with various edge densities and angle orientations and who found that graphene surfaces with a 37^o^ orientation have a potent antimicrobial impact against *S. aureus* and *P. aeruginosa*, while a surface orientation of 90^o^ was proven to possess the maximum bactericidal influence. Furthermore, they suggested that a graphene nanofilm surface does not act as a simple blade but, rather, causes pore formation within the bacterial cell membrane, resulting in osmotic disruption and, as consequence, cell death. Conversely, other findings suggest that GMs’ antimicrobial effects are determined by the availability of the basal planes rather than the edges of these nanosheets. In this regard, Mangadlao et al. (2015) performed an experiment in which they eliminated the GO edge effect by embedding them in polyethylene terephthalate (PET) and observed that GO did not lose its antimicrobial activity against *E. coli* when the edge effect was unavailable. Furthermore, they observed that the greater the number of basal planes, the better the antimicrobial activity. Based on this information, it can be supposed that the mechanical action of edges might not be required and that the basal planes of GO are considered the active sites in which the antibacterial effects of GO can be compromised by masking these basal planes through non-covalent adsorption (Hui et al., 2014).

### Oxidative Stress With or Without ROS Production

Generally, oxidative stress takes place through either a ROS-dependent or a ROS-independent pathway which, in either case, disrupts the cellular functions and mechanisms resulting in cellular inactivation and death. ROS-dependent oxidative stress occurs as a result of excessively cumulated intracellular ROS such as hydroxyl radicals (OH^•^), hydrogen peroxide (H_2_O_2_), singlet molecular oxygen (^I^O_2_), and superoxide anions (O_2_^• –^). These intracellular accumulated ROS induce cell membrane degeneration, lipid peroxidation, protein inactivation, mitochondrial dysfunction, and cell necrosis (West and Marnett, 2006). The ROS-independent pathway induces cell oxidation and disruption without ROS production; this may occur through charge transfer from the cell membrane to GMs, where graphene acts as an electron pump (Li et al., 2015).

#### ROS-Dependent Oxidative Stress

Reactive oxygen species production is considered a principal contributor to the antimicrobial activity of GMs (Gurunathan et al., 2012). GM-mediated ROS production starts from O_2_ adsorption on the GMs’ edges and defective sites, which subsequently undergo reduction through a variety of cell-enzymatic reactions such as interaction with glutathione (GSH) (Perreault et al., 2015a, b; Liu X. et al., 2011; Lu et al., 2017), α-tocopherol (Lu et al., 2016), or N-acetyl cysteine (NAC) (Gurunathan et al., 2012). GSH, as a significant antioxidant compound, is considered an indicator of the intracellular redox state because, in the presence of ROS, GSH can undergo oxidation into glutathione disulfide (GSSG); its depletion indicates the cytotoxic impact related to oxidative stress generated inside the involved bacterial cell (Gurunathan et al., 2012; Musico et al., 2014; Perreault et al., 2015a; Romero-Vargas Castrillón et al., 2015). Interestingly, the high number of oxygen-containing functional groups such as − OH and − COOH on the GMs’ surface enhances ROS production and prompts the antimicrobial activity of graphene particles. This statement was proven by Musico et al. (2014), who observed that increased ROS generation results in more oxidative stress to *B. subtilis* and *E. coli*. Furthermore, Gurunathan et al. (2012) proved the cytotoxic effects of ROS produced by GO and rGO against *E. coli*. it was proven that GM-induced ROS accumulation, intracellularly, is significantly related to mitochondrial membrane depolarization and dysfunction (Li et al., 2012). Also, ROS-induced cellular lipid oxidation results in the formation of lipid peroxide radicals, which cause progressive cell membrane damage and, subsequently, bacterial death (Jana et al., 1990; West and Marnett, 2006). However, it is crucial to perform additional investigations to obtain a better understanding of GM-bacteria interactions and their related pathways.

#### ROS-Independent Oxidative Stress

Despite the favorable concept of a ROS-mediated antimicrobial mechanism, not all researchers accept it. Scientists have continued their attempts to better explore various oxidative stress pathways, supposing that GMs’ oxidative capacity could be related to the high conductivity of these particles. In this regard, it was supposed that rGO’s capacity to oxidize GSH originates from the much higher conductivity of rGO in comparison to GO, in which rGO serves as a conductive bridge across the insulating lipid bilayer to intercede in the process of electron transmission from the bacterial intracellular environment to the external ambiance (Liu S. et al., 2011; Chen J. et al., 2013). debatable report, proposed by Li et al. (2015), supposed that GMs’ antimicrobial mechanism originates from electron transfer and not from ROS. Thus, they investigated the antimicrobial activity of GMs against *E. coli* and *S. aureus*, utilizing a graphene sheet on a Cu conductor, Ge semiconductor, and SiO_2_ insulator. They found that bacterial growth was inhibited in association with G-Cu and G-Ge films while G-SiO_2_ exhibited the opposite results. This contradiction was explained by the electron transfer theory, which is explained as follows: circuit formation enables electrons to transfer from the bacterial cell membrane to the graphene sheet and then to the underlying conductor or semiconductor (Cu and Ge, respectively) substrate while these electrons do not transfer to the underlying insulator (SiO_2_) substrate. This means that GMs serve as electron acceptors which pump electrons away from the bacterial cell membrane. These boosts both the theory of ROS-independent oxidative stress as well as the graphene surface interaction rather than ROS or edge-dependent mechanisms of GMs’ antimicrobial activity (Zou et al., 2016).

### Wrapping/Trapping Bacterial Membrane by the Thin, Flexible GM Sheets

As mentioned previously, graphene is the thinnest known material sheet, composed of a single layer of sp^2^-bonded carbon atoms in a honeycomb (hexagonal crystalline) structure. This structure gives graphene the property of unique flexibility, enabling it to act as a barrier which wraps and isolates bacteria from the circumferential environment. As with any other living organism, bacteria need specific physicochemical and nutritive conditions to survive; when these conditions are interrupted, the bacterial cell may die. Thus, GMs, with their unique wrapping property, have a significant antibacterial impact. Regarding this mechanism, the Rodrigues group found that GO exhibits potential antibacterial activity against both Gram-positive (*Rhodococcus opacus* and *Bacteriodes subtilis*) and Gram-negative (*Cupriavidus metallidurans* and *E. coli*) via wrapping the bacteria and inhibiting their proliferation (Mejías Carpio et al., 2012). Furthermore, other studies observed the perturbation of the bacterial cell membrane induced by GMs’ wrapping/trapping. In this regard, Chen J. et al. (2014) scanning electron microscopy (SEM) results revealed that GO sheets wrap/trap bacterial cells (*Pseudomonas syringae* and *Xanthomonas campestris* pv *undulosa*) to cause bacterial membrane perturbation. Moreover, their findings revealed bacterial cell membrane damage as a result of membrane depolarization due to the wrapping effect. Supporting these findings, the three-dimensional porous GO membrane, designed by Kanchanapally et al. (2015) with an approximately 300 nm pore size, demonstrated bactericidal activity against *S. aureus* due to mechanical wrapping/trapping with consequent cell membrane damage. Interestingly, several studies were carried out to correlate the GMs’ sheet size with their antimicrobial activities. Dallavalle et al. (2015) simulations to investigate the interaction models between a bacterial cell membrane and graphene nanosheets that range between 0.9 and 13.3 nm demonstrated that graphene nanosheets greater than 5.2 nm in size were capable of partially wrapping the bacterial surface via the intense hydrophobic interaction between lipid bilayers on a bacterial membrane and graphene sheets. This interaction leads to an undermined bacterial membrane. On the other hand, graphene nanosheets of less than 5.2 nm in size were capable of penetrating the cell membrane rather than wrapping. Similarly, Eda and Chhowalla (2010) result demonstrated the capability of large GO nanosheets to easily wrap cells, blocking the membrane’s active sites and inhibiting cellular proliferation. In contrast, smaller GO nanosheets were not capable of completely wrapping the bacterial surface; hence, the bacterial cell remains able to interact with the ambiance and realize its survival requirements. Several other researchers, however, are not convinced by the wrapping/trapping antibacterial mechanism (Akhavan et al., 2011; Liu S. et al., 2011). Thus, much effort is needed to conduct this mechanism to other GMs’ related factors that can modify their cytotoxic influence, such as particle size, material concentration, exposure duration, pH, and cell type.

### Self-Killing Effect

Interestingly, the interaction between bacteria and GMs reveals bacteria’s ability to reduce graphene. Akhavan and Ghaderi (2012) findings show that when viable bacteria were incubated with GO-nanosheets, those bacteria interacted with GONPs, decreased their oxygen-containing functional groups by glycolysis interaction. Those bacterially reduced GO nanosheets demonstrated further inhibition of bacterial proliferation in comparison to GO nanosheets that are not bacterially reduced. Another study demonstrated the capability of the marine bacteria *Shewanella* to reduce GO via a bacterial respiratory process under both aerobic and anaerobic conditions (Salas et al., 2010; Jiao et al., 2011; Wang G. et al., 2011). Similarly, *E. coli* were also shown to be capable of reducing CuO to CuO_2_ during bacterial inactivation (Paschoalino et al., 2008). This bacterial phenomenon was referred to as the self-killing effect because bacteria seem to be passively killing themselves while reducing GMs-NPs (Zou et al., 2016). The mechanism of the self-killing effect is still unclear; further investigation is required to illustrate the underlying pathway.

## Advantages of GMs Use

Despite the increasing demand of medical devices due to the increase in the age of the population, the number of patients experiencing infections after undergoing implants is constantly growing. This evidence is mainly due to the inefficacy of most of the anti-infective therapies that are commonly applied to counteract bacteria device colonization.

First of all, it has been largely demonstrated that most of the pathogens recovered around implants are resistant to antibiotics (Aslam et al., 2018); due to their fast and effective ability to adapt to the environment changes, bacteria developed many mechanisms to escape drugs effect. Accordingly, drug-resistant bacteria are able to pump out drugs by efflux pumps and to produce enzymes dedicated to the active principles inactivation; moreover, most of the antibiotics were developed some decades ago, thus they are not anymore targeted to the correct binding site as bacteria evolved their genome as a protective tool (Aslam et al., 2018).

Metallic ions such as silver has been also largely applied as antibacterial tool to their ability to perturbate bacteria membrane and interference with DNA replication. However, the extensive use of such ions also for daily tools such as topic creams and toothbrushes speeded up bacteria resistance; so, literature reported numerous examples where pathogens were able to counteract also metallic ions by modifying the outer membrane to inhibit ions penetration or by specialize efflux pump to bind and pump out metals (Maillard and Hartemann, 2013; Hobman and Crossman, 2015). Moreover, by increasing the dosage of silver to enhance its efficacy, strong side effects can occur such as irreversible pigmentation in the skin and the eyes, organ damages such as liver and kidney, irritation in the respiratory and intestinal tract (Stensberg et al., 2011).

Nitric oxide (NO) was also proposed as antibacterial tool with promising results toward the treatment of infected wounds (Mowbray et al., 2008), however, a prolonged exposure to NO was demonstrated to strongly activate the inflammatory cascade thus causing severe side effect due to this disproportionate immune response (Mowbray et al., 2008).

On the opposite, until today the use of GMs seems to be exempt from these side effects thus providing some important advantages in comparison to the previously mentioned strategies as summarized in Table 3.

**TABLE 3:** Most promising advantages and disadvantages of GMs applications.

Advantages	Disadvantages	References
**(1) Conductivity**: GMs can act as a superior electrical conductivity enables as a supercapacitor to power up the biomedical devices such as wearable or implantable devices	The presence of GMs can cause an imbalance of the environment such as pH lowering and causing inflammatory response	Hamzah et al., 2017; Reina et al., 2017; Huang et al., 2019
**(2) Mechanical**: GMs can confer excellent mechanical properties which enable sustained proliferation, proper adhesion an enhanced differentiation for hard tissue such as bone	The presence of GMs can modify the physical-chemical properties of the bulk material thus influencing its response to the environment	Reina et al., 2017; Priyadarsini et al., 2018
**(3) Antibacterial**: GMs hold a wide-range activity and can be used for both antibacterial and antiviral application	The GMs activity is not targeted toward specific receptors or pathways, so resistance can be developed by bacteria after long exposure	Ye et al., 2015; Yang et al., 2017; Valentini et al., 2019; Xia et al., 2019
**(4) Detection**: GMs ultra-sensitivity can strongly enhance biosensors efficacy for thermal or optical signals detection	GMs ultra-sensitivity can somehow interfere with detected signals and their use get up a lot the productive costs	He et al., 2010; Mo et al., 2015; Peña-Bahamonde et al., 2018
**(5) Water decontamination**: GMs can be used for industrial water treatment to remove ions bacteria and other contaminants	GMs are more effective than other decontamination agents but much more expensive	Wei et al., 2018

Moreover, GMs have been recently proposed as antiviral materials to fight some difficult viral infections. Firstly demonstrated that GO and rGO exhibit broad-spectrum antiviral activity toward both the DNA of Pseudorabies Virus (PRV) and to the RNA of porcine epidemic diarrhea virus (PEDV) at a nanocytoxic concentrations (Ye et al., 2015). Furthermore, Yang et al. (2017) looked at β-cyclodextrin functionalized graphene oxide and its possible role in combating respiratory syncytial virus (RSV), suggesting that the curcumin loaded functional GO was a highly efficient inhibitor of RSV infections maintaining cytocompatibility toward mammalian cells. Also showed that hypericin loaded onto graphene oxide (GO/HY) hold antiviral activity against Novel duck reovirus (NDRV), both *in vitro* and *in vivo* (Zheng et al., 2017; Zhou, 2004; Zou et al., 2016).

## Graphene Alloys

Although several mechanisms underlying the antimicrobial activities of metallic ions are not yet fully understood, metal ions are known for their potential antibacterial influence against both Gram-positive and Gram-negative bacteria. Attempts have been made to enhance the antibacterial impact of various metallic ions by conjugating different metals together to construct a hybridized alloy that maintains the desirable antibacterial properties of every single metallic component. Some alloy examples are discussed below:

### Silver-GMs Alloys

Silver nanoparticles (AgNPs) are well known for their broad-spectrum antimicrobial activity, and many studies have demonstrated their antibacterial efficacy against a variety of bacterial strains including *E. coli, S. aureus*, methicillin-resistant *S. aureus* (MRSA), and methicillin-resistant *Staphylococcus epidermidis* (Mansor et al., 2009; O’Hanlon and Enright, 2009; Lara et al., 2010). This antibacterial activity was explained by three possible mechanisms that ultimately result in bacterial cell death: (i) AgNPs-cell membrane direct contact, which results in increased permeability and damages the cell membrane, (ii) AgNPs and Ag-induced reactive oxygen species (ROS) production, or (iii) disturbed DNA replication and ATP production (Dakal et al., 2016). Furthermore, the bactericidal impact of Ag ions is influenced by the particle size; it has been confirmed that the smaller the AgNPs, the greater the antimicrobial activity (Matijevic, 1993; Khanna and Subbarao, 2003; Leopold and Lendl, 2003; Baker et al., 2005).

Because antimicrobial activity significantly affects the entire structure of the nanoparticles, a novel antibacterial system was introduced by GO-Ag hybrid composite construction (Anandan et al., 2010; Lightcap et al., 2010; Das et al., 2011; Liu L. et al., 2011). This composite demonstrates enhanced antimicrobial properties due to the modification of GO nanosheets’ surface by Ag, leading to better dispersion and stability of GONPs, in addition to the negative surface charge of the conjugated GO-Ag that reduces the bacteria-bacteria cell interaction (Dakal et al., 2016; Ma et al., 2011).

Different linking materials were used to conjugate AgNPs to GO sheets. Zhu et al. utilized diallyl-dimethyl-ammonium chloride (PDDA) to attach AgNPs to GO. The resulting GO-PDDA-AgNPs composite demonstrated significantly increased antibacterial activity in comparison to AgNPs alone (Dakal et al., 2016). Polyethyleneimine (PEI) is another linker material that Cai et al. used to produce a GO-PEI-AgNPs hybrid with antibacterial activity based on the blade-like edge which is characterized by long-term antibacterial activity and excellent stability and, consequently, causes bacterial cell destruction (Cai et al., 2011; Kooti et al., 2018). In another study, Cai et al. (2012b) conjugated AgNPs to sodium 1-naphthalenesulfonate functionalized reduced graphene oxide (NArGO); the resulting AgNP-NA-rGO nanostructure hybrid demonstrated extensive antibacterial capability, tremendous stability, and minor cytotoxicity. These conjugation approaches show significant enhancement in the antimicrobial activity by merging two potent antimicrobial materials, which is promising in different biomedical applications.

### GO-TiO_2_ Alloys

Titanium dioxide (TiO_2_) is a well-known semiconductor generally applied to industrial waste detoxification as a photocatalyst utilizing light energy (Fujishima and Honda, 1972). This compound has been employed in a diversity of applications. In addition to the safety outcomes of TiO_2_ for both ecological and human applications, as well as its high stability, it reveals significant bactericidal and bacteriostatic activities via its intense oxidative capacity and super hydrophilicity (Ditta et al., 2008; Ghosh and Das, 2015). As a photocatalytic antibacterial system, the antimicrobial activity can be derived using various mechanisms (Linsebigler et al., 1995): (i) electron-hole pair generation on the TiO_2_ surface when the excitation wavelength moves to the visible light (Fu et al., 2005; Li et al., 2005), (ii) the reaction of photo-generated holes with the adsorbed H_2_O or -OH, producing highly reactive hydroxyl radicals, and the response of electrons to oxygen to produce superoxide ions, and (iii) the destruction, by active oxygen species, of microorganisms attached to the TiO_2_ surface via oxidation activity (Linsebigler et al., 1995). Moreover, the photocatalytic capability of TiO_2_ can be enhanced by many strategies including particle size and/or surface modification by the addition of other semiconductors or metal nanoparticles (Sakthivel et al., 2004; Li et al., 2005; Liu et al., 2009). Thus, GO layers were modified with TiO_2_ to produce a nanocomposite that comprises the properties of both TiO_2_ and carbon (Han et al., 2015). Despite the desirable characteristics that the TiO2-GO nanocomposite showed, including clearness, conductivity, absorptivity, and controllability (Chang, 2013), it demonstrated cytotoxic effects toward some human cell lines such as adenocarcinomas human- alveolar basal epithelial cells (A549). These damaging effects were derived by the entrance of TiO_2_-GO nanoparticles into the cell, causing the mitochondrial injury which results in increased lysosome numbers as well as cellular disruption and damage (Jin et al., 2014).

### GO-ZnO Alloys

Zinc oxide (ZnO) is one of the distinct nanoparticles known for its high stability, high surface area to volume ratio (Sawai, 2003; Sawai and Yoshikawa, 2004; Huang et al., 2008; Jones et al., 2008; Tam et al., 2008), biosafety, low toxicity, and remarkable antibacterial activities against Gram-positive and Gram-negative bacteria and bacterial spores. Thus, in the medical research field, ZnONPs was introduced as a suitable drug delivery system, as well as in the cosmetic products field as an active ingredient (Rosi and Mirkin, 2005). Interestingly, ZnONPs demonstrate a superior antimicrobial impact against *S. aureus* in comparison to other metallic oxides, as reported by Jones et al. (2008). Another study carried out by Huang et al. (2008) explained that ZnO has a considerable antibacterial effect against *Streptococcus agalactia* and *S. aureus* by increasing the cell wall/membrane permeability and, thereby, bacterial cell disruption. Once these nanoparticles invade the bacterial cell wall/membrane, the antibacterial mechanism may take place through two pathways: (i) ROS production (Franklin et al., 2007; Lipovsky et al., 2009; Yang et al., 2009) and (ii) the interruption of the membrane and cellular functions as a result of NPs’ accumulation on the bacterial surface, inside the bacterial cytoplasm, or at the periplasmic space (Xu and Wang, 2009; Liu et al., 2010; Zhou et al., 2011; Wang et al., 2012, Wang Y.W. et al., 2014). The antimicrobial influence of ZnO can be affected by a variety of factors – mainly, the particle shape and size (Baruah and Dutta, 2009), UV light, aqueous suspension (Thill et al., 2006), and its concentration and hybridization with other NPs (Franklin et al., 2007; Lipovsky et al., 2009; Yang et al., 2009). Recently, ZnO and GO NPs was incorporated to construct a ZnO-GO nanocomposite that holds outstanding properties enabling it to be exploited in a variety of fields, including optics, sensors, electronics, and catalysts, as well as, more interestingly, targeted drug carriers and antibacterial tools (Xu and Wang, 2009; Liu et al., 2010; Zhou et al., 2011; Wang et al., 2012; Wang Y.W. et al., 2014). According to Wang and Cao’s investigations, it has been reported that ZnO-GO nanoparticles manifest high bactericidal activity against *E. coli* as well as low cytotoxic effects toward HeLa cells in different concentrations (Baruah and Dutta, 2009).

### GO-Gold Alloys

Because gold nanoparticles (AuNPs) are biocompatible, they have been significantly applied in the biomedical field for gene and drug delivery and photothermal therapy, as a contrast enhancer, and in biosensor technology (Skrabalak et al., 2008). The particles’ physical and chemical properties differ based on the particle shape manifestation and the size reduction to a nanometer level. The nanostructure of gold particles can be greatly influenced by the synthetic technique applied. Gold nanorods (GNR) were found to be favorable candidates for exterminating both *S. aureus* and *Propionibacterium acnes*, which are involved in follicular and dermatological diseases (Mahmoud et al., 2017). Biosynthesized AuPNs demonstrate optimistic activity against many pathogenic bacteria in which these NPs destroy the bacterial cell membrane via intracellular ROS production. Eventually, cell membrane disrupts due to the ROS-derived membrane lipid peroxidation that results in bacterial death (Zada et al., 2018). To enhance the antibacterial activity of AuNPs, they are bio-conjugated with various NPs, including GO, to produce AuNPs-GO and AuNPs-rGO hybrids that demonstrate high antimicrobial activity. The bactericidal activity of Au-GO nanocomposites was investigated by He et al. (2013) to reveal its antimicrobial potential against Gram-positive or Gram-negative bacteria and fungus following 2 h of irradiation under solar light. Furthermore, Hussain et al. (2014) decorated an Au-rGO nanocomposite; this study showed high bactericidal activity against some Gram-positive and Gram-negative bacteria as well as good biocompatibility in association with HeLa cells.

### GO-CuO Alloys

For a long time, copper has been known as a highly potent antimicrobial agent that rapidly kills bacteria, viruses, and yeasts through a process termed “contact killing,” i.e., the microorganism is killed when it comes into contact with the metallic copper surface (Grass et al., 2011). Because copper has been safely used as an antimicrobial agent in medical applications, it has recently been recorded with the United States Environmental Protection Agency (EPA) as the first solid antimicrobial material (Grass et al., 2011).

As copper particles became reduced to nano-size (CuNPs), they exhibit a highly toxic effect against most microorganisms in which cellular damage is derived by the CuNPs’ redox properties that cause lipid and protein oxidation and, ultimately, cell destruction (Yoshida et al., 1993; Faúndez et al., 2004). To avoid the cytotoxic effects of CuNPs in humans, it is crucial to increase the CuNPs’ stability and to control the Cu^2+^ release. To realize these goals, different substances were conjugated as copper carriers, such as titanium oxide (Sunada et al., 2003), silicon dioxide (Kim et al., 2006; Singh et al., 2010), activated carbon fiber (Byeon et al., 2007), zeolite (Top and Ülkü, 2004), phosphate glass fiber (Abou Neel et al., 2005), and montmorillonite (Hu and Xia, 2006). To improve the long-term antibacterial activity and water solubility of CuNPs, poly-l-lysine/reduced graphene oxide/copper nanoparticles (PLL-rGO-CuNPs) were produced in which PLL-rGO served as a carrier of CuNPs that were anchored on the rGO surface. The choice of this hybrid composition relied on the desirable properties of both PLL and rGO. PLL is characterized by its flexible framework structure, good biocompatibility, and favorable water solubility (Shan et al., 2009). Besides, as mentioned previously, rGO exhibits extreme potency as an antibacterial agent (Akhavan and Ghaderi, 2010; Hu et al., 2010). Moreover, previous studies have reported that rGO-based hybrids show superior antibacterial activity, desirable water solubility, and mild cytotoxicity (Cai et al., 2011). Thus, the PLL-rGO-CuNPs demonstrated a long-term additively antibacterial impact against Gram-negative *E. coli* and Gram-positive *S. aureus* in which 99.9% of bacteria were killed and the antibacterial mechanism was carried on throughout disruption of the ion concentrations of intracellular fluid, all of which indicate potential in microbial control applications (Ouyang et al., 2013; Yousefi et al., 2017). Additionally, copper dioxide nanoparticles (CuONPs) exhibit significant physical and chemical properties comprising antibacterial and antiviral activities (Gabbay et al., 2006; Borkow et al., 2009). This antibacterial activity against both Gram-positive and Gram-negative bacteria is derived from CuONPs’ ability to increase the permeability of the bacterial cell membrane followed by cell membrane disruption, accumulation of these nanoparticles inside the cell, and bacterial cell death as a consequence (Suleiman et al., 2013). The mechanism of this process relies on oxidative stress, genotoxicity, nano-hemostasis, and coordination influence that results in microorganism death (Chang et al., 2012).

### GO-Fe Alloys

Iron oxides, in particular Fe_2_O_3_ nanoparticles, are well known for their biological applications including biological tissue detoxification, tissue repair, and magnetic resonance imaging (MRI) resolution improvement (Amiri et al., 2012). Fe_2_O_3_NPs bactericidal activity was reported against *P. aeruginosa, E. coli*, and *S. aureus* (Yousefi et al., 2017). Fe_2_O_3_NPs with a bandgap of ≅2.2 eV exhibit bacterial inhibition as a result of their visible light absorption properties (≅564 nm) and unique magnetic properties (Long et al., 2017). Although these nanoparticles show moderate antibacterial activities against both Gram-positive and Gram-negative pathogenic bacteria, their antibacterial mechanism is still not clear. Similar to other metallic oxides, the antibacterial activity of Fe_2_O_3_NPs is particle-size-dependent and increases when the particle size is less than 50 nm. However, small nanoparticles can easily accumulate to form larger aggregates due to their high surface energy, resulting in restricted antibacterial activity (Long et al., 2017). A nanocomposite system consisting of rGO-iron oxide nanoparticles (rGO-IONPs) was used to study the antibacterial activity toward *S. aureus*. The mechanism of interaction between rGO-IONPs and bacteria was dependent on the generated heat and the high amount of hydroxyl radicals. The near-infrared (NIR) spectroscopy investigations demonstrated a noticeable reduction in bacterial cell viability both *in vitro* and *in vivo* (Pan et al., 2016). In another study, GO surface doped with Fe_2_O_3_ and AgNPs (MGO-Ag) displayed high antibacterial activity against *E. coli* and *S. aureus* where the viability decreased as 99.99 and 99.96%, respectively. Generally, the involved antibacterial mechanism is based on physical pressure, oxidative stress, and ROS production exerted by the applied nanoparticles (Feng et al., 2000; Morones et al., 2005; Tran et al., 2010; Yousefi et al., 2017).

## GMs Biomedical Application

Graphene and its derivatives have gained multidisciplinary interests in biomedical research because of their unique physio-chemical properties. Development of new methods of GMs synthesis has made this material much easier accessible in today’s market and gained more interesting in biomedical applications such as antimicrobial agents for tooth and bone implants, anticancer therapy, biofunctionalization of protein and in drug delivery application (Hamzah et al., 2017; Reina et al., 2017; Priyadarsini et al., 2018; Valentini et al., 2019).

Over the past few years, numerous new therapies and devices based on different materials have been discovered for the treatment of several diseases (Gillespie et al., 1988). Metallic devices such as stainless steel and other alloys have used in implants due to their mechanical properties. However, there are some disadvantages regarding their use due to exogenous toxicity or the lack of cellular adhesion (Huang et al., 2003).

Accordingly, GMs can be used in combination with other materials to improve their cytocompatibility and to introduce antibacterial properties as prior discussed.

As an example, GMs was successfully used in combination with hydroxyapatite to improve mechanical and pro-osteogenic properties of bone healing dedicated ceramics (Liu et al., 2014).

In the Dentistry field, GMs can be used to improve the mechanical properties of polymers used for reconstructive procedures: in fact, these materials hold poor resistance toward mechanical stress due to chewing compression, thus fast degrading and releasing toxic compounds. The use of GMs was successful in enhancing polymers mechanical resistance, thus prolonging their lifespan and reducing the release of toxic compounds coming from the degradation (Rosa et al., 2013).

Another example is related to smooth muscle repair. Here, GMs were successfully applied in combination with nitinol, an alloy made of nickel and titanium, to improve its cytocompatibilty resulting in enhancing cells proliferation (Shradhanjali et al., 2017).

Therefore, GMs have been shown to be effective in ameliorating the repair performance of different bulk materials for several biomedical applications.

## Conclusion and Future Perspectives

In summary, we presented recent advances and knowledge about graphene and its derivates for biomedical application with a particular focus on antibacterial properties and mechanisms. Graphene materials show a promising perspective in a variety of fields such as electronics, thermotics, biomedical applications and many others. In the Biomedical sector, GMs revealed a potent capacity to be involved in diagnostics, drug delivery, tissue engineering and infection control domains.

Many works proven that GMs hold high antibacterial activity against both Gram-positive and Gram-negative bacteria but depending on a variety of mechanisms and factors related to both, the bacterial components and the nanoparticle’s characteristics themselves. This review highlighted those factors including structural and physical-chemical properties, topographical features and material dispersion to offer a general view for further studies design. Also, the discussion about bacteria related factors including cellular components such as lipids, proteins and nucleic acids (DNA/RNA), as well as the bacterial self-killing mechanism, can offer the view of potential mechanism suitable to design MGs-based antibacterial tools.

However, many aspects are still worthy of further investigations to better understand GMs relation with bacteria and human body. In particular, much evidence is required to clearly understand and establish graphene antibacterial activity and to correlate this activity to the innate immune system as promising future applicable biomaterials for simultaneous tissue rehabilitation and potential substitution/or reduction of antibiotic usage.

## Author Contributions

HM and LR wrote the main part of the manuscript. MC and XZ greatly contributed to the graphene alloys part. EB made major contributions particularly in choosing the figures. YA-H, MA, and AC made a significant contribution at the revision stage. AK prepared and formulated the references and was responsible for manuscript revision.

## Conflict of Interest

The authors declare that the research was conducted in the absence of any commercial or financial relationships that could be construed as a potential conflict of interest.
